# Gender differences in the prevalence and impact factors of hysterical tendencies in adolescents from three eastern Chinese provinces

**DOI:** 10.1186/s12199-018-0695-2

**Published:** 2018-02-07

**Authors:** Qinglin Cheng, Li Xie, Yunkai Hu, Jinfeng Hu, Wei Gao, Yongxiang Lv, Yong Xu

**Affiliations:** 1 Department of Adolescents and Children’s Health, School of Public Health, Medical College of Soochow University, Suzhou, Jiangsu 215123 China; 2 Hangzhou Center for Disease Control and Prevention, Hangzhou, China; 3 Fuyang New Rural Cooperative Medical Office, Fuyang, China; 4 Shangcheng District Center for Disease Control and Prevention, Hangzhou, China; 5 Mingguang Health and Family Planning Commission, Mingguang, China; 6 Jin’an District Center for Disease Control and Prevention, Lu’an, China

**Keywords:** Hysterical tendencies, Adolescent, Multicenter case control study, Prevalence, Impact factors

## Abstract

**Background:**

Few studies have attempted to compare the differences in the prevalence and impact factors of hysterical tendencies (HTs) in adolescents. Thus, the aim of this study was to examine gender differences in the prevalence and impact factors of adolescents’ HTs across three eastern Chinese provinces (Anhui, Jiangsu, and Zhejiang).

**Methods:**

A multicenter, school-based, cross-sectional study was conducted in three provinces (Anhui, Jiangsu, and Zhejiang) in China in 2014. The sample included 10,131 middle-school students aged 13–18 years who were randomly selected using a multiphase, stratified, cluster sampling technique. A two-stage appraisal procedure was used to determine the adolescents’ HTs. We also designed a multicenter, school-based, case control (1329 cases with 2661 control individuals) study to collect data on the common factors affecting this population using a common protocol and questionnaire.

**Results:**

An overall positive rate of HTs among adolescents across the three eastern Chinese provinces studied was found at 13.1% (95% confidence interval (CI) 12.5–13.8%), at 14.5% (95% CI 13.3–15.7%) for females, and at 12.2% (95% CI 11.1–13.4%) for males. Gender-stratified, multiple conditional regression analyses revealed that superstitious beliefs pertaining to life, somatotype, teacher–student satisfaction, and family achievement orientation were significantly linked to HTs only in males, while left-behind adolescents, emotional and social adaptation, teacher–student support, family cohesion, and the Hospital Anxiety and Depression Scale - depression scores were significantly associated with female HTs only. The models indicated that of all the independent variables studied, family medical history was the strongest impact factor for both male HTs (adjusted matched odds ratio (amOR) = 2.92, 95% CI = 1.84–4.86) and female HTs (amOR = 2.74, 95% CI = 1.59–4.98).

**Conclusions:**

HTs are prevalent among adolescents in the three eastern Chinese provinces studied. Gender differences in the prevalence and impact factors of HTs are significant in adolescents, and HTs seem to affect more females than males. Therefore, sex-specific intervention programs against HTs in adolescents should be considered to reduce HT prevalence in adolescents by modifying influential social, school, and family factors.

## Background

Previous studies explained that hysterical phenomena are associated with diseases (e.g., paralysis or a loss of sensation) for which no organic lesion can be found, and, for which, in many cases, a pathological cause seems unlikely. Hysteria also referred to as *conversion disorder*, *psychogenic disorder*, *non-organic disease*, *functional disease*, *medically unexplained disease*, and *dissociative and somatoform disorder* [1–3].

Hysteria is one of the most prevalent diseases globally. Hysteria has a comparable prevalence among different countries (England, Switzerland, Finland, Turkey, the Netherlands, Germany, Italy, the USA, and China) [4–6], at 1–4% in the general population and 10% or more within psychiatric settings. Hysteria was found to be present in 14% of 25,018 respondents with post-traumatic stress disorder in 16 countries according to the World Health Organization (WHO) World Mental Health Surveys in 2012 [7]. Studies demonstrate that hysteria has major impacts on functioning and quality of life and often occurs in early life and for sustained periods, thereby resulting in many disease years [3]. Therefore, hysteria largely affects public health and results in high societal costs.

In recent decades, there has been a growing awareness of hysteria in childhood and adolescence [8–10]. Previous studies reported that the prevalence of hysteria was 9.1% in German adolescents (aged 14–24 years old) in 1995 [11]. Further, epidemiological studies of children and adolescents conducted in India found higher prevalence rates of hysteria [12] compared with western countries. In a literature study, we also found that youngers, the female gender, rural settings, low education levels, low socioeconomic statuses, marital status, having a family member with the disease, and childhood sexual abuse are associated with an increased risk of hysteria [13–15]. Kokota et al. showed that hysteria may result in significant social, economic, and health burdens in children and adolescents [8, 16]. Thus, hysteria is prevalent among adolescents. To reduce future hysteria prevalence and harm, it is necessary to explore the potential impact factors, including potentially modifiable ones, associated with hysteria.

Some studies suggested that young people show early signs before the onset of hysteria, such as hysterical tendencies (HTs) or hysterical personality (HP) traits [17–19]. According to Halleck et al., HTs are egoistic, immature, labile, extreme, and histrionic behavioral tendencies exhibited by individuals over a significant period of time [17, 18]. HTs result from preexisting personality traits that are defined by high hysteria scores [18, 20]. Some studies have indicated that HTs put individuals at risk for several adverse outcomes, including mass hysteria [17–21] and a range of other mental health problems, such as anxiety and depression [22, 23]. The links between HTs and adverse outcomes are thought to reflect the effects of the early signs of hysteria overtime. Even so, little information about HT examinations has been shared. For example, although the links between HTs and adverse outcomes have been well documented, no specific prevalence nor risk estimates are available pertaining to adolescent HTs [2, 24]. To investigate the prevalence and impact factors of HTs in a particular population, ideally, all influential factors of HTs should be ascertained. However, because HTs are difficult to measure, many studies include only a descriptive or single factor analysis. Little research has been conducted in terms of empirical investigations of gender-specific characteristics pertaining to HTs. Moreover, scarce theoretical work concerning the pathological mechanisms of HTs exists. In stark contrast, hysteria has been researched extensively pertaining to gender. To effectively decrease adolescents’ hysteria risk and prevalence rates, the potential impact factors of sex-specific HTs should be investigated using large-scale epidemiological studies in order to develop effective interventions against HTs.

The present study mainly focuses on the gender-specific prevalence and impact factors of HTs in adolescents. We hypothesized that, to the extent that it is possible to identify the environmental factors that are associated with HTs, environmental variables might mediate the association between gender and HTs. Thus, we designed a multicenter, cross-sectional study to discuss gender differences in terms of HT prevalence in adolescents across three eastern Chinese provinces (Anhui, Jiangsu, and Zhejiang). Then, a multicenter case control study was conducted to explore the gender-specific impact factors of HTs in this population.

## Methods

### Study design

**Fig. 1:**
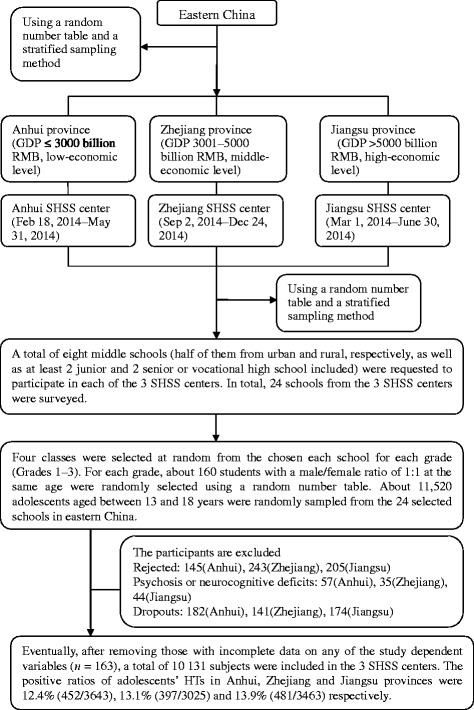
Data sampling. The prevalence of adolescents’ HTs did not seem to be connected with their economic levels (*P* > 0.05). *GDP* gross domestic product, *SHSS* School Health Surveillance System, *HTs* hysterical tendencies

This research was a school-based, cross-sectional, multicenter study in which schools were the unit of randomization. Schools were selected using a random number table and a stratified cluster sampling method of three School Health Surveillance System (SHSS) centers from three provinces (Anhui, Jiangsu, and Zhejiang) in China; in total, 24 schools were recruited. A sample of 480 students aged 13–18 years at each of the enrolled schools were randomly selected using a random number table and a stratified sampling method. To be included, adolescents had to be mainstream students (i.e., had no intellectual disability) aged 13–18 years and had to be able to converse in Chinese. Adolescents were excluded if they reported a history of psychosis or neurocognitive deficits or if they were receiving a secondary mental health service. Further details are available in Fig. 1.

We assessed HTs in adolescents using a two-stage appraisal procedure. In the first stage, HTs were screened by the Chinese version of Minnesota Multiphasic Personality Inventory (MMPI-2) Hysteria Scale, which includes 60 items [25, 26]. In the second stage, individuals whose responses to the Hysteria Scale suggested that they might have HTs were further assessed by three psychiatrists in order to obtain a final diagnosis. About 60 trained fieldworkers administered two questionnaires to subjects that were assessed as having HTs. Face-to-face assessments and investigations were conducted in each of the three SHSS centers by the fieldworkers. In addition, five trained fieldworkers were present in each of the three SHSS centers in order to control the quality of the research process.

We also designed a school-based, collaborative case control study; each center used a common protocol and core questionnaire. For each case of HTs, two control individuals matched for age and gender were randomly selected from the relevant SHSS center census database using a random number table method. Three or four control individuals were randomly selected to compensate for non-response and to ensure balance in the number of cases and control individuals at each center. To be included, control individuals had to be mainstream students (i.e., had no intellectual disability nor a history of psychosis nor neurocognitive deficits), have a uniform MMPI-2T-score < 60, be from the same area as the student studied from the case group, be the same sex and age as the student studied from the case group, and be mentally healthy. In order to ensure the consistency of the inclusion and exclusion criteria, all control individuals were assessed by a psychiatrist. Trained fieldworkers evaluated the potential impact factors of HTs in the case and control students using standardized assessment instruments, including the Family Environment Scale (FES), the Chinese Adaptation Scale for Adolescents (CASA), the Student–Teacher Relationship Scale (STRS), the Chinese version of the Peer Relationship Inventory (CPRI), and the Hospital Anxiety and Depression Scale (HADS).

### Measures and procedure

#### Adolescents’ HTs measurement

This study designed a two-stage appraisal procedure to identify the adolescents’ HTs. In the first stage, HTs were screened by the Chinese version of the MMPI-2 Scale, which includes 60 items [25, 26]. The MMPI-2 scale is a broadly applied, self-reporting hysteria scale. Respondents’ selections range from zero to one point for each item. The MMPI-2 raw score ranges from zero to 60, wherein a higher score marks a greater frequency and number of symptoms of hysteria. Originally, this scale was designed to measure the psychiatric symptoms of adults and adolescents (≥ 16 years old). In previous studies, however, when the scale was applied to children and youth samples, it was found to have satisfactory reliability and validity [27–29]. Therefore, the MMPI-2 scale was used to measure hysteria scores in this study in order to ascertain HTs. Internal consistency in the present sample was 0.89 (Cronbach’s coefficient). The inventory was scored using computer software. The raw scores of the scale were converted to a uniform T-score according to the transformation rule [30]. Then, the responses were summed.

Individuals whose responses to the MMPI-2 scale suggested that they might have HTs (i.e., high hysteria scores (with a uniform T-score ≥ 60) were further assessed by three psychiatrists (including two chief physicians and a senior attending doctor) to obtain a final diagnosis. The procedure was as follows: First, three psychiatrists carefully reviewed the diagnostic criteria of hysteria as well as related studies on HTs. Second, they excluded all patients with hysteria and other mental or personality disorders using the Diagnostic and Statistical Manual of Mental Disorders and the Chinese Classification of Mental Disorders. Third, they independently estimated HTs in participants using a semi-structured questionnaire that included questions on demographic characteristics and HP traits [17–19, 31, 32]. Furthermore, to ensure the consistency of the assessments of the different psychiatrists, a mutual evaluation form was used; thus, adolescent HTs were only confirmed when HTs were consistently diagnosed by all three psychiatrists. During this evaluation, the psychiatrist was primarily responsible for the diagnosis and differential diagnosis of HTs. HTs were noted if a subject (a) had a high hysteria score (a uniform T-score ≥ 60) [31, 32]; (b) had HP traits(i.e., egoistic, immature, labile, extreme, and histrionic behavior) [17, 18, 20]; (c) had precluding organic diseases, hysteria, or other mental or personality disorders; (d) had no history of psychosis nor neurocognitive deficits; and (e) had intact intellectual functioning (i.e., an intelligence quotient (IQ) > 70 according to the Wechsler Adult Intelligence Scale (WAIS-R) [33] in adolescents aged 17–18 years and the Wechsler Intelligence Scale for Children (WISC-IV) [34] in adolescents aged 13–16 years).

#### HTs as independently estimated by psychiatrists

A semi-structured questionnaire with 35 self-reported items (including demographic characteristics and HP traits) was designed to assess the HTs of the participants. The reliability of the questionnaire was tested on 56 individuals in Hangzhou, Zhejiang, with HTs not included in this study. The responses were analyzed using Cronbach’s alpha. The results revealed an internal consistency of 0.83 in terms of demographic characteristics and an internal consistency of 0.87 in terms of HP traits, suggesting the validity of the designed questionnaire.

#### IQ measurement

To assess the IQs of the participants, we administered the Chinese revision of WAIS-R in adolescents aged 17–18 years and the Chinese revision of the WISC-IV in adolescents aged 13–16 years. The Chinese revisions of WAIS-R and WISC-IV (including vocabulary, digit span, and digit symbol substitution subtests) are the most widely used and well-respected measures of general intelligence in China [33, 34]. The IQ tests were age standardized with a mean of 100 and a standard deviation of 15 in the general population [35]. Adolescents were assessed individually under the same standardized laboratory conditions at all ages. In the present study, intact intellectual functioning was assessed on a full scale [33, 34]. The field workers were trained to a uniform standard, and all of the tests were scored by two independent testers.

#### Demographic characteristic measurement

We designed a questionnaire to assess the demographic factors that contribute to HTs. The questionnaire included three main aspects: individual characteristics (gender, age, left-behind adolescents, and somatotype (referring to the overall outlook of the body to convey the totality of morphological features of the human body)), family factors (family income, family size, and superstitious beliefs in life (regarding a broad range of constructs from the belief in psychic powers to beliefs that are not attributable to scientific or religious explanations, such as supernatural, paranormal, or otherwise magical beliefs)), family medical history (e.g., history of psychosis or neurocognitive diseases in the family), and school characteristics(e.g., boarding school, administration model, and class size).

#### Social adjustment status measurement

The CASA is comprised of three factors: emotional adaptation (6 items), social adaptation (6 items), and study and life adaptation (5 items) [36]. Related research suggested that the CASA has high reliability and validity, with a Cronbach’s coefficient of 0.80 [36, 37]; thus, the CASA can be used as an effective tool to assess adolescents’ adaptation for the social life. Adolescents’ adaptation is discussed in four points (with a total score from 17 to 68). In the present sample, the internal consistency was 0.88.

#### School environment status measurement

##### Teacher–student relationship

The STRS is a 28-item self-report instrument designed to measure a teacher’s perception of her or his relationship with a target student [38]. The Chinese version of the STRS (STRS-CV) is the translated and revised version of the STRS by Qu [39] based on Pianta’s STRS [38]. It includes four domains: intimacy, conflict, support, and satisfaction. Good internal consistency and acceptable test–retest reliability (with a Cronbach’s coefficient of 0.71–0.87) were present in related research [39]. The teacher–student relationship was tested using a five-point Likert scale for a total score of 23–115 points. In the present sample, the internal consistency was 0.87.

##### Peer relationship

The CPRI is a 20-item self-report instrument designed to assess an adolescent’s peer relationship. The CPRI was revised by Wei et al. [40] based on the 13-item Missouri Peer Relations Inventory [41]. The CPRI measures three analytically derived dimensions of peer relations: social maturity, aggression, and independence. The scale has high reliability and validity (with a Cronbach’s coefficient of 0.58–0.86) [40]. The peer relationship was analyzed using a five-point Likert counts for a total score of 20–100 points. In the present sample, the internal consistency was 0.90.

##### Family environment status measurement

The FES is a 90-item self-report instrument used to measure relationships, personal growth, and system maintenance in the context of one’s family environment [42]. The Chinese version of the FES (FES-CV) was amended by Phillips [43]. Previous studies found that 10 subscales of the FES-CV demonstrated moderate to excellent internal consistency (ranging from 0.63–0.75) and acceptable test–retest reliability at 0.55–0.92 [43]. In the present sample, the internal consistency was 0.85.

##### Assessment of anxiety and depression

Anxiety and depression were evaluated using the HADS [44]. The HADS was specifically developed to detect anxiety and depression in patients with somatic conditions. It is divided into an anxiety subscale (HADS-A) and a depression subscale (HADS-D), both of which contain seven items with scores from zero to three, giving a possible maximum score of 21. Scores less than eight indicate no clinical distress, scores from eight to 10 point out possible psychiatric morbidity, and scores ≥ 11 suggest probable pathologic levels of distress [44]. Good internal consistency and acceptable reliability of the Chinese versions of the HADS have been demonstrated (with Cronbach coefficients of 0.81 for anxiety and 0.83 for depression) [45, 46]. In the present sample, the internal consistency was 0.86.

### Statistical analysis

All data were entered twice, verified, and de-identified in the EpiData open-source database (version 3.1). We analyzed the data using R version 3.2.2. The imputation of the missing data was conducted separately for each center, and three imputed datasets were created.

We used descriptive statistics to calculate the frequencies and percentages of the categorical variables as well as the mean ± standard deviation of the normally distributed continuous variables. The matched odds ratio (mOR), adjusted mOR (amOR), and a 95% confidence interval (CI) were used to measure the associations between impact-factors and adolescents’ HTs.

We used univariate analyses to separately select all the covariates (i.e., demographics and environment factors) that were potentially associated with HTs in order to enter them into multiple regression models at a significance level of 0.05. Independent *t* tests were used for the continuous variables with a normal distribution, Bartlett’s One-factor Analysis of Variance was used for three or more variables with a normal distribution and homogeneity of variance, and the Pearson’s chi-squared test and Kruskal–Wallis nonparametric test were employed to identify equilibrium between cases and controls.

We used a conditional multiple logistic regression model to evaluate whether the covariates, including demographics, environmental factors, and individual personality, were associated with HTs. A stepwise procedure was used to further select the covariates that were associated with HTs at a significance level of *P* > 0.10 for removal and a significance level of *P* < 0.05 for reentry. The final model was selected according to the minimum statistics of the Akaike information criterion. Hypothesis testing was conducted using a two-sided test with an alpha value of 0.05 to indicate statistical significance.

## Results

### Baseline characteristics of the subjects

A response rate of 89.4% was obtained. In total, 10,294 questionnaires were completed at 24 schools. After removing questionnaires with incomplete data in any of the study’s dependent variables (*n* = 163, accounting for 1.6%), 10,131 participants were retained for subsequent analyses. The overall positive rate of HTs among the eastern Chinese adolescents was 13.1% (1330/10,131, 95% CI = 12.5–13.8%), at 14.5% (95% CI = 13.3–15.7%) for females and 12.2% (95% CI = 11.1–13.4%) for males. The positive rate of HTs in females was higher than that in males (chi-square = 7.04, *P* = 0.008, mOR = 1.22, 95% CI = 1.05–1.41).

We enrolled a total of 1329 HT cases and 2661 control individuals in our multicenter, case control study. Table 1 presents the central locations and dispersions of the uniform T-scores among the different baseline characteristics of all the subjects. Statistical differences in the uniform T-score were only found in terms of gender (in the cases and controls). The case and control subjects were matched by age and gender (1:2 pair-wise matching) (Table 1).

**Table 1:** Baseline characteristics of all subjects in the multi-center case-control study

Variables	Total (*N* = 3990)	HTs	Controls	*P* value	mOR (95%CI)	Uniform T-score of MMPI-2 Hysteria Scale (*M* ± SD)			
		(*n* = 1329)	(*n* = 2661)			HTs (*n* = 1329)	*P* value	Controls (*n* = 2661)	*P* value
Age (years)									
13	858 (21.5)	286 (21.5)	572 (21.5)	1.000^a^	NA	67.0 ± 5.0	0.272^c^	47.0 ± 8.5	0.102^c^
14	810 (20.3)	269 (22.2)	541 (20.3)			66.5 ± 4.8		46.8 ± 9.0	
15	723 (18.1)	241 (18.1)	482 (18.1)			66.2 ± 5.0		46.7 ± 8.5	
16	660 (16.5)	220 (16.6)	440 (16.5)			66.2 ± 4.5		47.0 ± 8.5	
17	474 (11.9)	158 (11.9)	316 (11.9)			66.6 ± 4.7		46.6 ± 9.1	
18	465 (11.7)	155 (11.7)	310 (11.7)			66.0 ± 4.9		47.5 ± 8.4	
Gender									
Male	1842 (46.2)	614 (46.2)	1228 (46.1)	1.000^b^	1.00(0.88–1.14)	66.3 ± 4.7	**< 0.001** ^*d*^	46.8 ± 8.7	**0.012** ^*d*^
Female	2148 (53.8)	715 (53.8)	1433 (53.9)			67.6 ± 5.2		47.5 ± 8.9	
Nationality									
Han	3943 (98.8)	1315 (98.9)	2628 (98.8)	0.604^b^	1.18(0.64–2.19)	66.4 ± 4.8	0.333^d^	46.3 ± 8.4	0.834^d^
Ethnic minorities	47 (1.2)	14 (1.1)	33 (1.2)			67.7 ± 5.7		46.6 ± 8.7	
Residence area									
Rural	2157 (54.1)	730 (54.9)	1427 (53.6)	0.419^b^	1.06(0.93–1.21)	66.8 ± 4.9	0.056^d^	46.7 ± 8.8	0.475^d^
Urban	1833 (45.9)	599 (45.1)	1234 (46.4)			66.2 ± 4.7		46.5 ± 8.4	
Sampling area									
Anhui	1354 (33.9)	451 (33.9)	903 (33.9)	0.999^a^	NA	66.5 ± 4.9	0.802^c^	46.7 ± 8.5	0.892^c^
Zhejiang	1195 (29.9)	397 (29.9)	798 (30.0)			66.6 ± 4.8		46.5 ± 8.8	
Jiangsu	1441 (36.2)	481 (36.2)	960 (36.1)			66.3 ± 4.7		46.7 ± 8.7	

Data expressed as *n* (%). Uniform T-score according to formula conversion methods for standardization. Uniform T-scores of HTs and control groups are compared respectively in baseline characteristics. Bold values are those that reach statistical significance (*P* < 0.05)

*M* ± SD mean ± standard deviation, *HTs* hysterical tendencies, *MMPI* Minnesota Multiphasic Personality Inventory, *mOR* matched odds ratios, *CI* confidence interval, *NA* not available

^a^Kruskal-Wallis nonparametric tests

^b^Pearson chi-square test

^c^Bartlett’s one-way ANOVA

^d^Independent *t* tests

### Gender differences in terms of the impact factors of HTs

Tables 2 and 3 summarize the results of the univariate analyses of the association between an individual covariate and HTs. About 20 of the 37 tested covariates were significantly associated with male HTs only (*P* < 0.05). The significant covariates were (a) demographic characteristics, including school administration model, family medical history, and superstitious beliefs; (b) social adjustment characteristics in terms of emotional adaptation; (c) teacher–student relationships, including intimacy, support, and satisfaction; (d) peer relationships; (e) family environment characteristics, including cohesion, expressiveness, and conflict; and (f) psychological factors according to the HADS-A. The remaining 17 covariates, including family income, education level and occupation of parents, boarding school, class staff, number of family members, left-behind adolescents, social adaptation, study and life adaptation, teacher–student conflict, peer interpersonal interaction and harmony, family active–recreational orientation and control, and the HADS-D score, did not significantly relate to male HT risk (*P* > 0.05) (Tables 2 and 3). In contrast, about 23 covariates, including left-behind adolescents, social adaptation, teacher–student conflict, family active–recreational orientation, and the HADS-D score, were significantly associated with female HTs (Tables 2 and 3).

**Table 2:** Gender-specific for comparison of demographic characteristics between HTs and controls (*N* = 3990)

Variables	Males				Females			
	HTs (*n* = 614)	Controls (*n* = 1228)	*P* value^a^	*mOR* (95%CI)	HTs (*n* = 715)	Controls (*n* = 1433)	*P* value^a^	*mOR* (95%CI)
Family income								
Below middle level	121 (19.7)	220 (17.9)	0.351	1.12 (0.87–1.45)	115 (16.1)	188 (13.1)	0.063	1.27 (0.98–1.64)
Middle level and above	493 (80.3)	1008 (82.1)			600 (83.9)	1245 (86.9)		
Education level of father								
High school and below	521 (84.9)	1065 (86.7)	0.273	0.86 (0.65–1.14)	602 (84.2)	1153 (80.5)	**0.035**	**1.29 (1.01–1.66)**
College degree and above	93 (15.1)	163 (13.3)			113 (15.8)	280 (19.5)		
Education level of mother								
High school and below	548 (89.3)	1116 (89.3)	0.265	0.83 (0.60–1.17)	644 (90.1)	1276 (89.0)	0.467	1.12 (0.82–1.52)
College degree and above	66 (10.7)	112 (10.7)			71 (9.9)	157 (11.0)		
School administration model								
Fully-closed	187 (30.5)	440 (35.8)	**0.013**	NA	205 (28.7)	425 (29.7)	**0.002**	NA
Semi-closed	311 (50.6)	611 (49.8)			378 (52.9)	824 (57.5)		
Open	116 (18.9)	177 (14.4)			132 (18.4)	184 (12.8)		
Father’s occupation								
Peasants, no professions	115 (18.7)	221 (18.0)	0.701	1.05 (0.81–1.36)	174 (24.3)	321 (22.4)	0.316	1.11 (0.90–1.38)
Other	499 (81.3)	1007 (82.0)			541 (75.7)	1112 (77.6)		
Mother’s occupation								
Peasants, no professions	185 (30.3)	375 (30.5)	0.858	0.98 (0.79–1.22)	244 (34.1)	498 (34.8)	0.765	0.97 (0.80–1.18)
Other	429 (69.7)	853 (69.5)			471 (65.9)	934 (65.2)		
Recent environment pollution								
Yes	267 (43.5)	470 (38.3)	**0.031**	**1.24 (1.01–1.52)**	245 (34.3)	480 (33.5)	0.722	1.03 (0.85–1.26)
No	347 (56.5)	758 (61.7)			470 (65.7)	953 (66.5)		
Superstitious beliefs in life								
Yes	134 (21.8)	175 (14.3)	**< 0.001**	**1.68 (1.30–2.17)**	108 (15.1)	194 (13.5)	0.325	1.14 (0.87–1.48)
No	480 (78.2)	1053 (85.7)			607 (84.9)	1239 (66.5)		
Boarding school								
Yes	149 (24.3)	314 (25.6)	0.543	0.93 (0.74–1.17)	221 (30.9)	371 (25.9)	**0.014**	**1.28 (1.04–1.57)**
No	465 (75.7)	914 (74.4)			494 (69.1)	1062 (74.1)		
Class staff								
Yes	244 (39.7)	524 (42.7)	0.229	0.89 (0.72–1.08)	386 (54.0)	820 (57.2)	0.154	0.88 (0.73–1.05)
No	370 (60.3)	704 (57.3)			329 (46.0)	613 (42.8)		
Family medical history								
Yes	40 (6.5)	27 (2.2)	**< 0.001**	**3.10 (1.83–5.31)**	43 (6.0)	25 (1.7)	**< 0.001**	**3.60 (2.13–6.21)**
No	574 (93.5)	1201 (97.8)			672 (94.0)	1408 (98.3)		
Number of family members								
> 5	271 (44.1)	555 (45.2)	0.667	0.96 (0.78–1.17)	303 (42.4)	654 (31.7)	0.152	0.88 (0.73–1.05)
≤ 5	343 (55.9)	673 (54.8)			412 (57.6)	779 (68.3)		
Class size								
> 45	353 (57.5)	597 (48.6)	**< 0.001**	**1.43 (1.17–1.75)**	369 (51.6)	642 (44.8)	**0.003**	**1.31 (1.09–1.58)**
≤ 45	261 (42.5)	631 (51.4)			346 (48.4)	791 (55.2)		
Left-behind adolescents								
Yes	154 (25.1)	265 (21.6)	0.091	1.22 (0.96–1.54)	215 (30.1)	316 (22.1)	**< 0.001**	**1.52 (1.23–1.87)**
No	460 (74.9)	963 (78.4)			500 (69.9)	1117 (77.9)		
Somatotype								
Ineligible	389 (63.4)	699 (56.9)	**0.008**	**1.31 (1.07–1.61)**	383 (53.6)	710 (49.5)	0.079	1.17 (0.98–1.41)
Eligible	225 (36.6)	529 (43.1)			332 (46.4)	723 (50.5)		

Data expressed as *n* (%). Bold values are those that reach statistical significance (*P* < 0.05)

*HTs* hysterical tendencies, *mOR* matched odds ratios, *CI* confidence interval, *NA* not available

^a^Pearson chi-square test

**Table 3:** Gender-specific for comparison of environmental factors between HTs and controls (*N* = 3990)

Variables	Males (*M* ± SD)			Females (*M* ± SD)		
	HTs (*n* = 614)	Controls (*n* = 1228)	*P* value^a^	HTs (*n* = 715)	Controls (*n* = 1433)	*P* value^a^
Social adjustment status						
Emotional adaptation	17.2 ± 3.9	17.7 ± 3.6	**0.012**	17.1 ± 3.6	17.8 ± 3.3	**< 0.001**
Social adaptation	16.9 ± 4.0	17.1 ± 3.5	0.239	17.0 ± 3.4	17.5 ± 3.3	**0.003**
Study and life adaptation	13.0 ± 3.9	13.1 ± 3.5	0.532	12.8 ± 3.5	13.5 ± 3.2	**< 0.001**
School environment status						
Teacher-student relationship						
Intimacy	18.4 ± 5.4	18.9 ± 5.3	**0.047**	18.3 ± 4.9	19.1 ± 4.9	**0.001**
Support	13.2 ± 3.8	14.1 ± 3.6	**< 0.001**	13.3 ± 3.6	14.3 ± 3.4	**< 0.001**
Satisfaction	14.6 ± 3.6	15.5 ± 3.3	**< 0.001**	14.7 ± 3.1	15.4 ± 2.9	**< 0.001**
Conflict	16.2 ± 6.0	15.8 ± 6.0	0.137	14.7 ± 5.6	13.7 ± 5.1	**< 0.001**
Peer relationship						
Social emotion	22.1 ± 5.9	22.8 ± 5.2	**0.014**	22.8 ± 5.4	23.0 ± 4.5	0.374
Interpersonal interaction	18.6 ± 5.5	18.9 ± 4.7	0.197	19.0 ± 4.7	18.9 ± 4.0	0.740
Interpersonal harmony	22.6 ± 6.6	23.0 ± 5.8	0.182	23.3 ± 6.1	23.3 ± 5.4	0.965
Family environment status						
Relationships dimensions						
Cohesion	6.1 ± 1.9	6.8 ± 2.0	**< 0.001**	6.4 ± 2.2	7.1 ± 2.0	**< 0.001**
Expressiveness	4.8 ± 1.6	5.0 ± 1.6	**0.007**	5.2 ± 1.7	5.4 ± 1.6	**0.022**
Conflict	3.6 ± 2.1	3.0 ± 2.0	**< 0.001**	3.5 ± 2.0	2.9 ± 1.9	**< 0.001**
Personal growth dimensions						
Independence	4.8 ± 1.7	5.2 ± 1.6	**< 0.001**	4.7 ± 1.6	5.1 ± 1.5	**< 0.001**
Achievement orientation	5.2 ± 1.6	5.8 ± 1.7	**< 0.001**	5.2 ± 1.7	5.6 ± 1.7	**< 0.001**
Intellectual-cultural orientation	4.0 ± 1.8	4.3 ± 1.9	*0.010*	4.3 ± 1.7	4.4 ± 1.9	0.777
Active-recreational orientation	4.6 ± 1.9	4.7 ± 2.0	0.408	4.7 ± 2.1	4.9 ± 2.1	*0.044*
Moral-religious emphasis	5.2 ± 1.5	5.5 ± 1.5	**< 0.001**	5.5 ± 1.5	5.8 ± 1.5	**< 0.001**
System maintenance dimensions						
Organization	5.7 ± 1.7	6.0 ± 1.8	**< 0.001**	5.8 ± 2.9	6.2 ± 1.8	**< 0.001**
Control	4.1 ± 1.7	4.2 ± 1.8	0.056	4.2 ± 1.9	4.2 ± 1.9	0.770
HADS-A score	5.9 ± 2.4	3.5 ± 1.6	**< 0.001**	7.8 ± 2.8	3.8 ± 2.3	**< 0.001**
HADS-D score	4.1 ± 2.0	3.9 ± 2.1	0.065	7.2 ± 2.6	3.6 ± 1.8	**< 0.001**

Bold values are those that reach statistical significance (*P* < 0.05)

*M* ± SD mean ± standard deviation, *HTs* hysterical tendencies, *HADS* hospital anxiety and depression scale, *HADS-A* HADS-anxiety, *HADS-D* HADS-depression

^a^Independent *t* tests

To further explore possible gender differences in the association between HTs and environmental factors, we performed gender-stratified, multiple regression analyses. Analysis stratified by gender showed that superstitious beliefs in terms of life, somatotype, teacher–student satisfaction, and family achievement orientation were significantly linked to HTs only in males, while left-behind adolescents, emotional and social adaptation, teacher–student support, family cohesion, and the HADS-D score were significantly associated with female HTs only. Four variables (family medical history, family conflict, family independence, and the HADS-A score) were related to HTs in both genders (Tables 4 and 5). The models also indicated that of all of the independent variables, family medical history was the strongest impact factor for both male HTs (amOR = 2.92, 95% CI = 1.84–4.86) and female HTs (amOR = 2.74, 95% CI = 1.59–4.98) (Tables 4 and 5).

**Table 4:** Analyses of conditional multiple logistic regression of male HTs on HT-associated environment factors

Variables	Coefficient	Standard error (coefficient)	Wald	*P* value	amOR (95% CI)
Demographic characteristics					
Age	− 0.0124	0.0319	− 0.39	0.698	0.99 (0.92–1.05)
School administration model	0.1399	0.0805	1.74	0.082	1.15 (0.98–1.35)
Recent environment pollution	0.0972	0.1077	0.9	0.367	1.10 (0.89–1.36)
Superstitious beliefs in life	0.3121	0.1394	2.24	0.025	**1.37 (1.04–1.80)**
Family medical history	0.6852	0.2731	5.89	< 0.001	**2.92 (1.84–4.86)**
Class size	0.2913	0.1023	1.79	0.071	1.34 (0.98–1.82)
Somatotype	0.2367	0.1093	2.16	0.030	**1.27 (1.02–1.57)**
Social adjustment status					
Emotional adaptation	0.0120	0.0153	0.79	0.432	1.01 (0.98–1.04)
School environment status					
Teacher-student relationship					
Intimacy	0.0240	0.0136	1.76	0.078	1.02 (0.99–1.05)
Support	− 0.0145	0.0189	− 0.77	0.442	0.99 (0.95–1.02)
Satisfaction	− 0.0670	0.0217	− 3.09	0.002	**0.88 (0.84–0.98)**
Peer relationship					
Social emotion	0.0014	0.0114	0.12	0.904	1.00 (0.98–1.02)
Family environment status					
Relationships dimensions					
Cohesion	− 0.0668	0.0348	− 1.92	0.054	0.94 (0.87–1.00)
Expressiveness	0.0042	0.0355	0.12	0.906	1.00 (0.94–1.08)
Conflict	0.2796	0.1297	2.68	0.007	**1.28 (1.02–1.59)**
Personal growth dimensions					
Independence	− 0.1256	0.0335	− 3.75	< 0.001	**0.82 (0.79–0.91)**
Achievement orientation	− 0.2410	0.0358	− 3.94	< 0.001	**0.73 (0.62–0.88)**
Intellectual-cultural orientation	− 0.0116	0.0307	− 0.38	0.706	0.99 (0.93–1.05)
Moral-religious emphasis	− 0.0215	0.0400	− 0.54	0.591	0.98 (0.90–1.06)
System maintenance dimensions					
Organization	0.0680	0.0371	1.83	0.067	1.07 (0.99–1.15)
HADS-A score	0.4032	0.1604	3.56	< 0.001	**1.89 (1.37–2.94)**

The parameter estimates for each demographic characteristic, social adjustment status, school, and family environment factors were controlled by gender. Bold values are those that reach statistical significance (*P* < 0.05)

*HTs* hysterical tendencies, *amOR* adjusted matched odds ratios, *CI* confidence interval, *HADS* hospital anxiety and depression scale, *HADS-A* HADS-anxiety

**Table 5:** Analyses of conditional multiple logistic regression of female HTs on HT-associated environment factors

Variables	Coefficient	Standard error (coefficient)	Wald	*P* value	amOR (95%CI)
Demographic characteristics					
Age	0.0016	0.0348	0.05	0.963	1.01 (0.94–1.08)
Education level of father	0.0648	0.0453	1.51	0.092	1.09 (0.96–1.32)
School administration model	0.1548	0.0791	1.96	0.050	1.17 (1.00–1.36)
Boarding school	0.0926	0.0232	1.38	0.118	1.05 (0.91–1.57)
Family medical history	0.7632	0.1964	5.37	< 0.001	**2.74 (1.59–4.98)**
Class size	0.0789	0.0518	− 1.82	0.067	0.91 (0.83–1.28)
Left-behind adolescents	0.4373	0.1127	3.88	< 0.001	**1.55 (1.24–1.93)**
Social adjustment status					
Emotional adaptation	− 0.0454	0.019	− 2.39	0.017	**0.96 (0.92–0.99)**
Social adaptation	− 0.0579	0.0217	− 2.67	0.008	**0.84 (0.71–0.91)**
Study and life adaptation	− 0.0213	0.0204	− 1.05	0.296	0.98 (0.94–1.02)
School environment status					
Teacher-student relationship					
Intimacy	0.0149	0.0137	1.09	0.275	1.02 (0.99–1.04)
Support	− 0.0494	0.0189	− 2.61	0.009	**0.88 (0.82–0.93)**
Satisfaction	− 0.0387	0.0225	− 1.72	0.086	0.96 (0.92–1.01)
Conflict	0.0065	0.0106	0.62	0.538	1.01 (0.99–1.03)
Family environment status					
Relationships dimensions					
Cohesion	− 0.1010	0.0329	− 3.07	0.002	**0.80 (0.75–0.86)**
Expressiveness	0.0340	0.0347	0.98	0.326	1.03 (0.97–1.11)
Conflict	0.0658	0.0289	2.28	0.023	**1.17 (1.10–1.23)**
Personal growth dimensions					
Independence	− 0.3256	0.1320	− 3.93	< 0.001	*0.72 (0.63–0.85)*
Achievement orientation	− 0.0608	0.0326	− 1.86	0.062	0.94 (0.88–1.00)
Active-recreational orientation	0.0032	0.0260	0.12	0.902	1.00 (0.95–1.06)
Moral-religious emphasis	− 0.0103	0.0362	− 0.28	0.776	0.99 (0.92–1.06)
System maintenance dimensions					
Organization	0.0045	0.0324	0.14	0.888	1.00 (0.94–1.07)
HADS-A score	0.5214	0.1866	4.39	< 0.001	**1.94 (1.43–3.87)**
HADS-D score	0.4998	0.1634	4.23	< 0.001	**1.87 (1.31–3.69)**

The parameter estimates for each demographic characteristic, social adjustment status, school and family environment factors were controlled by gender. Bold values are those that reach statistical significance (*P* < 0.05)

*HTs* hysterical tendencies, *amOR* adjusted matched odds ratios, *CI* confidence interval, *HADS* hospital anxiety and depression scale, *HADS-A* HADS-anxiety, *HADS-D* HADS-depression

## Discussion

To the best of our knowledge, this study was the first to investigate the prevalence of HTs among a large sample of Chinese adolescents and to explore the associations between HTs and a broad range of environmental factors. Our findings demonstrate that HTs are prevalent among adolescents in China. The results of this study are in line with those of previous studies that reported that the prevalence of dissociative tendencies in the population is between 10 and 19% [19]. Similar results have been found in some studies of hysteria, such as adolescents’ hysteria is prevalent in the world [47]. Based on our findings, it would be particularly beneficial to reduce HT prevalence in adolescents by modifying impact factors and individual characteristics to prevent hysteria.

In the present study, the prevalence of HTs in adolescents was higher in females than in males. The reason for this gender difference might be related to the differences in emotional development in adolescence. Our research suggested that females require greater social adjustment than boys, while support from teachers is a key determinant for females dealing with mental health problems. Furthermore, gender differences in the HT prevalence rate may reflect different genetic and/or environmental factors. As such, sex-specific intervention programs against HTs in adolescents should be implemented.

Currently, a standard approach to measure HTs remains unavailable, and the screening of HTs is somewhat subjective and dependent on the availability of good-quality data. This decreases the internal validity of our study. In our study, HT adolescents were identified using a two-stage appraisal procedure in which both subjective and objective evaluation methods were integrated; this reduced subjectivity in our screening of HTs and correspondingly improved the internal validity of the study. In addition, since a large sample was used along with a multiphase, stratified, cluster sampling method, and a high response rate (89.4%), our study was powerful in detecting weak associations between outcomes and impact factors and significantly minimized possible selection bias.

In our study, left-behind adolescents, emotional and social adaptation, teacher–student support, family cohesion, and HADS-D scores contributed to female HTs only. HTs seem to affect more females than males. One possible reason is that males and females show certain disparities with biological and environmental factors implicated in the development of mental problems [48]. Even though HTs may be caused by biogenetic, environmental, and stress-related factors [17, 18, 20], male adolescents with good mental health can obtain social support through their personality traits and mutually beneficial interpersonal relationships to reduce the symptoms of HTs. Thus, they can gain support from interpersonal feedback, and their feelings of loneliness and helplessness are decreased; as such, HTs may be reduced. In addition, females are relatively more vulnerable to changes in psychological quality than males, and they are more easily influenced by social and family circumstances [49]. Research indicated that definitions of masculinity and femininity have psychological consequences for males and females, as they produce perceived gender differences, which includes differences in terms of stressors, experiences, coping strategies, social relationships, personal resources, and vulnerabilities [49–51]. For example, girls are more likely to use social relationships as avenues for self-disclosure, emotional intimacy, and support than males [52]. Evidence supports positive social relationships benefit women’s mental health more than men’s [53]. These findings are in agreement with our own.

Our findings also indicate that left-behind adolescents are only associated with female HTs. Parental migration results in left-behind adolescents being raised by other family members, such as grandparents [54]. Thus, the differences between parents and other caretakers in terms of family roles, education levels, and lifestyles may contribute to an unfavorable environment for the psychological development of adolescents’ mental health [55]. Further, in the present study, teacher–student support affects female HTs more than male HTs. This may be because adolescent girls report more interpersonal concerns than boys [56]. Studies show that the quality of teacher–student relationships in middle school has implications for adolescents’ future academic, social, and behavioral outcomes [57, 58]. In addition, teacher support may be especially important to female students’ engagement in middle school [59], when females are coping with stressful life events and when their independent coping skills are developing. A secure relationship with teachers may serve as a resource that permits young students to cope more effectively with novel academic and social demands. For instance, Frymier et al. reported that, among female adolescents, emotional security with the teacher attenuated adolescent stress reactivity to negative events in the classroom [60]. We also found that interpersonal relationships in the families of females with HTs are characterized by more conflict, less cohesion, and less independence, compared with the families of males with HTs. Further, a negative family climate may also be implicated in the maintenance of mental disorders [61, 62]. This is probably due to the complex and reciprocal influences of the overall family environment on the adaptation of female members. Numerous reports have discussed the correlation between conversion disorders and depressive moods in adolescents [63, 64]. In this study, HTs in adolescents were predicted by depressive moods using a categorical regression analysis. This matches the clinical observations of dissociative tendencies during adolescence reported by Evren et al. [65]. Further, it is well known that favorable growth environments in the family and at school are conducive to adolescent mental health. Our research also finds teacher–student satisfaction and family achievement orientation are only associated with male HTs. This association may be attributed to the personality traits of male adolescents (such as conscientiousness, openness, and extroversion). In monitoring HTs, male adolescents’ beliefs, families, and schools should be monitored. Furthermore, a focus on family education, adaptability, and teacher–student support is required for females.

Notably, family medical history, family conflict, family independence, and HADS-A scores are associated with both male and female HTs. Our study suggests that biogenetic, psychological, and family factors are key factors that affect adolescents’ risk of HTs. Becker-Blease et al. reported that genetic and environmental factors contributed to dissociative tendencies in children and adolescents [66]. This result is consistent with our own. There has also been an evidence to suggest that adolescents with anxiety are associated with an increased risk to develop mental disorders [67]. Adolescents’ HTs may be a serious public health problem in the future in East China. Therefore, the existence of HTs among adolescents should be emphasized as a high-risk psychological problem. Education and mental health professionals should observe high-risk adolescents for HTs. A positive family climate may provide a safe haven for high-risk adolescents, support healthy development and optimal learning and living, and discourage maladaptive behavior. Sheeber et al. found that stable family circumstances can play a key role in promoting the psychological health of adolescents [68]. Another study suggested that family conflict had unique and direct associations with the emotional health of adolescents [69]. Hence, mental therapy and family interventions are key to addressing adolescents at a high risk of HTs.

Most importantly, this study suggests that family medical history is the strongest impact factor for adolescent HTs. Some researchers found a highly significant association between family medical history and somatosensory amplification tendencies in adolescents [70]. This result is similar to the findings of our study. A family history of mental disorders is a significant impact factor for HTs in adolescents, as the association between family history and adolescent HTs was generally stronger for the cases than the controls in this study. WHO also indicated that each parent disorder examined, with the exception of suicide, was associated with an increased risk of every class of offspring mental disorder [71]. Although other studies found more specificity in the intergenerational transmission of particular disorders, including anxiety disorders [72] and major depression [73], the majority of these studies were based on relatively small samples. The large-scale, population-based nature of our study is unique and consequently may have produced results that are more generalizable than those of smaller and more select samples.

Several limitations of this study merit consideration. First, some data in our study were self-reported and were possibly underreported. However, the confidential nature of the questionnaire used in our study may have significantly decreased underreporting. Second, because no standard approach exists to measure HTs, misclassification of some outcomes may have resulted in the underestimation of the associations between HTs and impact factors. However, this may have been reduced by integrating both subjective and objective methods to measure HTs. Third, only adolescents attending school were sampled; out-of-school adolescents were not included in the study. Thus, generalizing the results from the study population to the entire adolescent population in China should be done cautiously.

## Conclusion

Our findings demonstrate that HTs are prevalent (13.1%) among adolescents across the three eastern Chinese provinces (Anhui, Jiangsu, and Zhejiang) studied. Adolescents’ HTs are influenced by multiple environments. Gender differences in terms of the impact factors of HTs in adolescents are very significant, and HTs seem affect females predominantly. As an effective measure of preventive individual and mass hysteria, early assessments and interventions for sex-specific HTs in adolescents play a significant role. In addition, the strongest association of a family’s medical history implies that intervening to treat any family mental disorder would likely reduce future HTs in adolescents. Effective interventions to prevent future HT cases would consequently require protecting the adolescent from the full range of family mental disorders. Given that family mental disorders are robust predictors of HTs in adolescents according to existing studies, it is important to recognize the potential public health importance of interventions to improve the functioning of mentally ill relatives and to reduce the prevalence of HTs in adolescents. Moreover, synthetic interventions of the individual, family, school, and society factors that result in HTs are needed. Finally, experimental research is required to determine how to improve adolescents’ psychological quality, social adaptability, and living and study environments and to explore whether these improvements would lead to greater resistance to stressful life events, decreased HTs, and enhanced health-promoting behaviors.

## Abbreviations

amOR: Adjusted matched odds ratio
CASA: Chinese Adaptation Scale of Adolescents
CI: Confidence interval
CPRI: Chinese version of the Peer Relationship Inventory
FES: Family Environment Scale
FES-CV: Chinese version of the FES
HADS: Hospital Anxiety and Depression Scale
HADS-A: Hospital Anxiety and Depression Scale (Anxiety)
HADS-D: Hospital Anxiety and Depression Scale (Depression)
HP: Hysterical personality
HTs: Hysterical tendencies
IQ: Intelligence quotient
MMPI: Minnesota Multiphasic Personality Inventory
mOR: Matched odds ratios
SHSS: School Health Surveillance System
STRS: Student–Teacher Relationship Scale
WAIS-R: Wechsler Adult Intelligence Scale
WISC-IV: Wechsler Intelligence Scale for Children

## References

[1] Abse DW (2013). Hysteria and related mental disorders: an approach to psychological medicine.

[2] Lovinger S (2006). Conversion hysteria in a pre-adolescent girl. Psychoanal Soc Work.

[3] Hofstra MB, Van Der Ende J, Verhulst FC (2002). Child and adolescent problems predict DSM-IV disorders in adulthood: a 14-year follow-up of a Dutch epidemiological sample. J Am Acad Child Adolesc Psychiatry.

[4] Coons PM (1998). The dissociative disorders. Rarely considered and underdiagnosed. Psychiatr Clin North Am.

[5] Sar V (2011). Epidemiology of dissociative disorders. An overview. Epidemiol Res Int.

[6] Mueller-Pfeiffer C, Rufibach K, Perron N, Wyss D, Kuenzler C, Prezewowsky C (2012). Global functioning and disability in dissociative disorders. Psychiatry Res.

[7] Stein DJ, Koenen KC, Friedman MJ, Hill E, McLaughlin KA, Petukhova M (2013). Dissociation in posttraumatic stress disorder: evidence from the world mental health surveys. Biol Psychiatry.

[8] Kokota D (2011). View point: episodes of mass hysteria in African schools: a study of literature. Malawi Med J.

[9] Ouss L, Tordjman E (2014). Conversive disorders among children and adolescents: towards new “complementarist” paradigms?. Clin Neurophysiol.

[10] Kozlowska K, Palmer DM, Brown KJ, Scher S, Chudleigh C, Davies F (2015). Conversion disorder in children and adolescents: a disorder of cognitive control. J Neuropsychol.

[11] Lieb R, Pfister H, Mastaler M, Witcchen H-U (2000). Somatoform syndromes and disorders in a representative sample of adolescents and young adults: prevalence, comorbidity and impairment. Acta Psychiatr Scand.

[12] Malhi P, Singhi P (2002). Clinical characteristics and outcome of children and adolescents with conversion disorder. Indian Pediatr.

[13] Obimakinde AM, Ladipo MM, Irabor AE (2015). Familial and socio-economic correlates of somatisation disorder. Afr J Prim Health Care Fam Med.

[14] Neeleman J, Ormel J, Bijl RV (2001). The distribution of psychiatric and somatic ill health: associations with personality and socioeconomic status. Psychosom Med.

[15] Scaife B, Gill PS, Heywood PL, Neal RD (2000). Socio-economic characteristics of adult frequent attenders in general practice: secondary analysis of data. Fam Pract.

[16] Sham FM, Hamjah SH, Ariff MI, Ismail R, Mohamed SN, Muhamat R (2012). A study of hysteria among youth in a secondary school in Malaysia. Adv Nat Appl Sci.

[17] Chodoff P, Lyons H (1958). Hysteria, the hysterical personality and “hysterical” conversion. Am J Psychiatry.

[18] Halleck SL (1967). Hysterical personality traits: psychological, social, and iatrogenic determinants. Arch Gen Psychiatry.

[19] Kihlstrom JF, Glisky ML, Angiulo MJ (1994). Dissociative tendencies and dissociative disorders. J Abnorm Psychol.

[20] Groth-Marnat G (2009). Handbook of psychological assessment.

[21] Chen CS, Yen CF, Lin HF, Yang P (2003). Mass hysteria and perceptions of the supernatural among adolescent girl students in Taiwan. J Nerv Ment Dis.

[22] Zoccolillo M (1992). Co-occurrence of conduct disorder and its adult outcomes with depressive and anxiety disorders: a review. J Am Acad Child Adolesc Psychiatry.

[23] Lazare A, Klerman GL (1968). Hysteria and depression: the frequency and significance of hysterical personality features in hospitalized depressed women. Am J Psychiatry.

[24] World Health Organization (WHO). Risks to mental health: an overview of vulnerabilities and risk factors. 2016. http://www.who.int/mental_health/mhgap/risks_to_mental_health_EN_27_08_12.pdf. Accessed 22 Mar 2016.

[25] Zhang J, Song W, Cheung FM (2004). The Chinese Minnesota Multiphasic Personality Inventory-2 (MMPI-2) (Chinese language edition).

[26] Cheung FM, Cheung SF, Zhang J (2004). Convergent validity of the Chinese Personality Assessment Inventory and the Minnesota Multiphasic Personality Inventory-2: preliminary findings with a normative sample. J Pers Assess.

[27] Archer RP (2015). Using the MMPI with adolescents.

[28] Espelage DL, Cauffman E, Broidy LM, Steiner H (2003). A cluster-analytic investigation of MMPI profiles of serious male and female juvenile offenders. J Am Acad Child Psy.

[29] Gumbiner J, Arriaga T, Stevens A (1999). Comparison of MMPI–A, Marks and Briggs, and MMPI–2 norms for juvenile delinquents. Psychol Rep.

[30] Tellegen A, Ben-Porath Y (1992). The new uniform t-scores for the MMPI-2: rationale, derivation, and appraisal. Psychol Assess.

[31] Ji SM, Dai ZS, Li MX (2004). Minnesota Multiphasic Personality Inventory––a new study and assessment of multiple scales.

[32] Yung YF, Chan W, Cheung FM, Leung K, Law JS, Zhang JX (2000). Standardization of the Chinese Personality Assessment Inventory: the prototype standardization method and its rationale. Asian J Soc Psychol.

[33] Gong Y (1992). The manual of Wechsler adult intelligence scale revised in China.

[34] Choi AL, Zhang Y, Sun G, Bellinger DC, Wang K, Yang XJ (2015). Association of lifetime exposure to fluoride and cognitive functions in Chinese children: a pilot study. Neurotoxicol Teratol.

[35] Breslau N, Paneth N, Lucia VC, Paneth-Pollak R (2005). Maternal smoking during pregnancy and offspring IQ. Int J Epidemiol.

[36] Zhang YB (2008). The current situation and relationship of self-cognition, self-evaluation and adaptation of Chinese junior high school students.

[37] Zhang DJ, Jiang Q (2006). A report on construction of adaptation scale for adolescent. Stud Psychol Behav.

[38] Pianta RC (2001). The Student–Teacher Relationship Scale.

[39] Qu ZY (2002). The characteristics of class environment and its relationship with students’ school adjustment in primary and middle school.

[40] Wei YH (1997). A study on structure model and factors influencing the self-esteem development in children and adolescents.

[41] Borduin CM, Ronis ST. Research note: Individual, family, peer, and academic characteristics of female serious juvenile offenders. Youth Violence Juv Justice. 2012;10:386–400.

[42] Moos RH, Moos BS (1981). Manual for the family environment scale.

[43] Phillips MR, West CL, Shen Q, Zheng Y (1998). Comparison of schizophrenic patients’ families and normal families in China, using Chinese versions of FACES-II and the family environment scales. Fam Process.

[44] Bjelland I, Dahl AA, Haug TT, Neckelmann D (2002). The validity of the Hospital Anxiety and Depression Scale: an updated literature review. J Psychosom Res.

[45] Leung CM, Wing YK, Kwong PK, Lo A, Shum K (1999). Validation of the Chinese-Cantonese version of the Hospital Anxiety and Depression Scale and comparison with the Hamilton Rating Scale of Depression. Acta Psychiatr Scand.

[46] Hong JS, Tian J (2014). Prevalence of anxiety and depression and their risk factors in Chinese cancer patients. Support Care Cancer.

[47] Carota A, Calabrese P (2014). Hysteria around the world. Front Neurol Neurosci.

[48] Zahn-Waxler C, Shirtcliff EA, Marceau K (2008). Disorders of childhood and adolescence: gender and psychopathology. Annu Rev Clin Psychol.

[49] Lewis AJ, Kremer P, Douglas K, Toumborou JW, Hameed MA, Patton GC (2015). Gender differences in adolescent depression: differential female susceptibility to stressors affecting family functioning. Aust J Psychol.

[50] Pearlin LI (2010). The life course and the stress process: some conceptual comparisons. J Gerontol B Psychol Sci Soc Sci.

[51] Thoits PA (2010). Stress and health major findings and policy implications. J Health Soc Behav.

[52] Moreira JFG, Telzer EH (2015). Changes in family cohesion and links to depression during the college transition. J Adolesc.

[53] Elliott M (2001). Gender differences in causes of depression. Women Health.

[54] Zhang N, Bécares L, Chandola T (2015). Does the timing of parental migration matter for child growth? A life course study on left-behind children in rural China. BMC Public Health.

[55] Gao Y, Li LP, Kim JH, Congdon N, Lau J, Griffiths S (2010). The impact of parental migration on health status and health behaviours among left behind adolescent school children in China. BMC Public Health.

[56] Xu Y, Farver JAM, Shin Y (2014). Shyness and psychosocial functioning in South Korean children. Eur J Pers.

[57] Wentzel KR (1998). Social relationships and motivation in middle school: the role of parents, teachers, and peers. J Educ Psychol.

[58] Cornelius-White J (2007). Learner-centered teacher-student relationships are effective: a meta-analysis. Rev Educ Res.

[59] Reddy R, Rhodes JE, Mulhall P (2003). The influence of teacher support on student adjustment in the middle school years: a latent growth curve study. Dev Psychopathol.

[60] Frymier AB, Houser ML (2000). The teacher-student relationship as an interpersonal relationship. Commun Educ.

[61] Reiss F (2013). Socioeconomic inequalities and mental health problems in children and adolescents: a systematic review. Soc Sci Med.

[62] Peris TS, Sugar CA, Bergman RL, Chang S, Langley A, Piacentini J (2012). Family factors predict treatment outcome for pediatric obsessive-compulsive disorder. J Consult Clin Psychol.

[63] Ani C, Reading R, Lynn R, Forlee S, Garralda E (2013). Incidence and 12-month outcome of non-transient childhood conversion disorder in the UK and Ireland. Br J Psychiatry.

[64] Pehlivantürk B, Unal F (1999). Conversion disorder in children and adolescents: clinical features and comorbidity with depressive and anxiety disorders. Turkish J Pediatr.

[65] Evren C, Can S (2007). Clinical correlates of dissociative tendencies in male soldiers with conversion disorder. Isr J Psychiatry Relat Sci.

[66] Becker-Blease KA, Deater-Deckard K, Eley T, Freyd JJ, Stevenson J, Plomin R (2004). A genetic analysis of individual differences in dissociative behaviors in childhood and adolescence. J Child Psychol Psychiatry.

[67] Cummings CM, Caporino NE, Kendall PC (2014). Comorbidity of anxiety and depression in children and adolescents: 20 years after. Psychol Bull.

[68] Sheeber L, Hops H, Alpert A, Davis B, Andrews J (1997). Family support and conflict: prospective relations to adolescent depression. J Abnorm Child Psychol.

[69] Timmons AC, Margolin G (2015). Family conflict, mood, and adolescents’ daily school problems: moderating roles of internalizing and externalizing symptoms. Child Dev.

[70] Köteles F, Freyler A, Kökönyei G, Bárdos G (2015). Family background of modern health worries, somatosensory amplification, and health anxiety: a questionnaire study. J Health Psychol.

[71] McLaughlin KA, Gadermann AM, Hwang I, Sampson NA, Al-Hamzawi A, Andrade LH (2012). Parent psychopathology and offspring mental disorders: results from the WHO World Mental Health Surveys. Br J Psychiatry.

[72] Eley TC, McAdams TA, Rijsdijk FV, Lichtenstein P, Narusyte J, Reiss D (2015). The intergenerational transmission of anxiety: a children-of-twins study. Am J Psychiatry.

[73] Hammen C, Hazel NA, Brennan PA, Najman J (2012). Intergenerational transmission and continuity of stress and depression: depressed women and their offspring in 20 years of follow-up. Psychol Med.

